# Pancreatic Cancer in Cystic Fibrosis: Is the Incidence Increasing?

**DOI:** 10.1002/ppul.71283

**Published:** 2025-09-09

**Authors:** Riley Markham, Brittany A. Wright, Tahuanty Pena

**Affiliations:** ^1^ University of Iowa Iowa City USA; ^2^ Department of Pharmacy University of Iowa Healthcare Iowa City Iowa USA; ^3^ Department of Internal Medicine Stead Family, Department of Pediatrics, University of Iowa Carver College of Medicine University of Iowa Healthcare Iowa City Iowa USA

**Keywords:** cystic fibrosis, malignancy, pancreatic cancer

## Author Contributions

**Riley Markham:** investigation, writing−original draft, writing − review and editing, formal analysis, methodology, data curation. **Brittany A. Wright:** conceptualization, writing − review and editing. **Tahuanty Pena:** conceptualization, formal analysis, writing − original draft, writing − review and editing, supervision, software, methodology.

## Conflicts of Interest

The authors declare no conflicts of interest.

To the Editor,

Cystic fibrosis (CF) is a genetic disease that affects multiple systems, due to mutations in the gene encoding the CF transmembrane conductance regulator (CFTR) protein. In the pancreas, the defective CFTR function disrupts ion transport within pancreatic ducts, leading to thickened secretions, ductal obstruction, chronic inflammation, and eventual fibrosis. Furthermore, obstruction of pancreatic ducts leads to impaired digestive enzymes release, resulting in pancreatic insufficiency [1].

Chronic inflammation is a well‐established risk factor for pancreatic cancer. In addition to chronic pancreatitis, other recognized risk factors include smoking, type 2 diabetes, family history of pancreatic cancer, obesity, and heavy alcohol use. Pancreatic cancer can be broadly categorized into two types: pancreatic adenocarcinoma, the most common form originating from exocrine cells, and pancreatic neuroendocrine tumors, which arise from endocrine cells and are relatively rare [2].

People with CF (pwCF) are at an increased risk of developing pancreatic cancer, with previous studies suggesting a five‐ to tenfold higher risk compared to the general population. This heightened susceptibility is believed to stem from chronic pancreatitis, due to CFTR dysfunction, compounded by other factors such as altered bile flow, oxidative stress, and changes in gut microbiota [3]. However, despite the increased relative risk, diagnosis of pancreatic cancer remains rare in pwCF, with an estimated incidence of ~1/100,000 cases per year [4]. This is significantly different from recent data on incidence of pancreatic cancer in the general population [5]. Current research on this association is limited by small sample sizes, incomplete data, and a lack of center‐specific studies.

This study aimed to evaluate the incidence of pancreatic cancer among pwCF cared for at our adult CF center. By utilizing patient data from a single tertiary center, we sought to characterize clinical factors associated with cancer risk, including CFTR mutation status, history of chronic pancreatitis, diabetes, and smoking history. We aimed to provide critical insights into the incidence of pancreatic cancer within a specific population, helping to address gaps in the literature and inform future screening and management strategies.

## 1 Methods

This study was approved by our Institutional Review Board (approval number 202412298) as exempted from informed consent due to minimal risk and conducted as a retrospective chart review using electronic medical records. The study population included individuals aged 18 years and older with a confirmed diagnosis of CF who received care at the University of Iowa Health Care between January 1, 2012, and January 1, 2025.

Incidence rates were calculated by dividing the number of new pancreatic cancer cases observed during the study period by the total number of person‐years at risk. The cohort grew from 63 individuals in 2012 to 179 individuals in 2024. Assuming linear population growth, the average population size was estimated at 121.0 individuals, yielding approximately 1573.0 person‐years of observation across the 13‐year study period. The crude incidence rate was expressed as the number of cases per 100,000 person‐years. To contextualize these findings, the calculated rate was compared against the national average reported by the Surveillance, Epidemiology, and End Results (SEER) Program (2023), and an incidence rate ratio (IRR) was derived.

Exact 95% confidence intervals for incidence rates were calculated using the exact Poisson method based on chi‐square distribution quantiles, and IRRs versus the SEER rate were derived with Poisson‐based confidence limits via the Ulm method. A one‐sided mid‐P exact test was then performed to assess whether the observed count of three cases differed from the SEER‐expected Poisson mean, with mid‐P values calculated according to Fay's approach.

In addition to incidence analysis, descriptive statistics were used to summarize demographic and clinical characteristics of the study population, including measures such as means, standard deviations, medians, and proportions. These descriptive data supported comparison between individuals with and without pancreatic cancer diagnoses. All statistical analyses were performed using Python (version 3.11).

## 2 Results

From January 1, 2012, to January 1, 2025, our cohort consisted of 179 pwCF (Table 1). The majority of pwCF, were male (51.4%), White (98.9%), pancreatic insufficient (82.1%), carried at least one copy of the F508del mutation and are receiving treatment with a CFTR modulator (91.1%). Of our cohort, 39.7% pwCF have a diagnosis of CF related diabetes (CFRD) based on a positive oral glucose tolerance test (OGTT) and 1.7% pwCF have another form of diabetes unrelated to their CF.

**Table 1:** Characteristics of the University of Iowa's Cystic Fibrosis cohort.

Cystic Fibrosis Cohort (179) Patients)	
*Age n (%)*	
Under 29 years old	54 (30.2%)
30−39 years old	53 (29.6%)
40−49 years old	36 (20.1%)
50 + years old	36 (20.1%)
*Sex: n (%)*	
Male	92 (51.4%)
Female	87 (48.6%)
*Race: n (%)*	
White	177 (98.9%)
Other	2 (1.1%)
*CFTR mutation: n (%)*	
Heterozygous F508del	82 (45.8%)
Homozygous F508del	81 (45.3%)
Other genotype	16 (8.9%)
*CF modulators: n (%)*	
Eligible for modulators	163 (91.1%)
Not eligible for modulators	16 (8.9%)
*Pancreatic insufficient: n (%)*	
Yes	147 (82.1%)
No	32 (17.9%)
*CFRD: n (%)*	
Yes	71 (39.7%)
Other diabetes	3 (1.7%)
No	105 (58.6%)
*Current FEV1 percent predicted: n (%)*	
Above 100%	54 (30.2%)
Between 50% and 99.99%	97 (54.2%)
Below 49.99%	24 (13.4%)
No PFT data	4 (2.2%)

Three pwCF in our cohort had pancreatic cancer. All patients had at least one copy of the F508del mutation. Patients 1 and 2 were males with Stage 4 Adenocarcinoma of the pancreas and passed away less than 2 months after diagnosis. Patient 3 is a female who was diagnosed with a Stage 2 neuroendocrine tumor in the pancreas. All three patients had CFRD and were pancreatic insufficient. None of the patients had a family history of pancreatic cancer, pancreatitis episodes, or ever smoked. Characteristics of all patient's treatment courses are summarized in Table 2. Two of these malignancies were exocrine pancreatic adenocarcinomas, whereas one was a pancreatic neuroendocrine tumor (pNET).

**Table 2:** Characteristics of cystic fibrosis patients with pancreatic cancer.

Pancreatic cancer cases among cystic fibrosis patients			
	Patient 1	Patient 2	Patient 3
CFTR mutation	F508del/G551D	F508del/F508del	F508del/D1152H
Sex:	M	M	F
Age at Diagnosis (year)	63	53	53
Days from diagnosis to death	48	50	n/a
Cancer treatment	None	Chemotherapy^a^	Surgery^b^
Tumor type	Adenocarcinoma	Adenocarcinoma	Neuroendocrine
Stage	4	4	2
CFRD	Yes	Yes	Yes
Pancreatic insufficiency	Yes	Yes	Yes
Family history of pancreatic cancer	No	No	No
Pancreatitis episode	Never	Never	Never
Smoking history	Never	Never	Never

a FOLFOX (Leucovorin calcium (folinic acid), Fluorouracil, Oxaliplatin);

b Whipple surgery at time of diagnosis.

The cohort population increased from 63 individuals in 2012 to 179 in 2024, with an estimated average population size of 121.0 individuals, resulting in approximately 1573.0 person‐years at risk. Based on this, and the three incident cases of pancreatic cancer diagnosed, the crude incidence rate of pancreatic cancer in the CF cohort was calculated to be 190.7 cases per 100,000 person‐years (95% CI 39.3−557).

## 3 Discussion

In this retrospective cohort study of adults with CF followed over a 13‐year period, we observed a crude incidence rate of 190.7 cases of pancreatic cancer per 100,000 person‐years. This figure is notably elevated compared to the current U.S. national incidence rate of 13.4 per 100,000 person‐years, as reported by the SEER Program in 2023 [5]. The IRR calculated from this comparison is 14.2 (95% CI 2.8−40.4; *p* = 0.0014), suggesting that pwCF in our cohort had more than 14 times the risk of developing pancreatic cancer compared to the general population.

Importantly, our cohort comprised both exocrine pancreatic adenocarcinoma (*n* = 2) and a pNET) (*n* = 1). Although SEER aggregates these histologies under a single category, their pathogenesis, risk factors, and natural history differ substantially, so comparisons should be interpreted with this caveat.

These findings are consistent with previous research demonstrating increased gastrointestinal cancer risk in individuals with CF. Maisonneuve and Lowenfels reported significantly elevated risks for digestive tract malignancies in a large‐scale meta‐analysis [6]. This is also demonstrated by Yamada and colleagues in a metanalysis from 2018 [7]. Similarly, Neglia et al. (2005) found that CF patients were at significantly increased risk—up to tenfold—for gastrointestinal and pancreatic cancers [8]. While pancreatic cancer is rare, its disproportionately high incidence in this CF population highlights the changing disease landscape as life expectancy improves due to advancements such as CFTR modulators.

The growing burden of malignancy in the aging CF population has also been highlighted in registry data, such as the UK CF Registry Annual Report (2020) [9]. Taken together, these findings suggest that routine cancer surveillance, particularly for pancreatic cancer, once developed, may become increasingly relevant in long‐term CF care. Although our sample size was limited, the magnitude of the observed incidence rate supports further investigation into targeted screening strategies and the identification of modifiable risk factors.

Limitations of this study include its retrospective, single‐center design and the small number of observed events, as well as lack of age adjustment. These constrain generalizability and statistical power. Nonetheless, the stark contrast with national incidence rates underscores the need for larger, multicenter epidemiological studies to guide evidence‐based screening protocols in CF populations.

## Data Availability

The data that support the findings of this study are available on request from the corresponding author. The data are not publicly available due to privacy or ethical restrictions.
